# Determining Diagnostic Sensitivity: A Comparison of Rose Bengal Test, Coombs Gel Test, ELISA and Bacterial Culture in Brucellosis Diagnosis—Analyzing Clinical Effectiveness in Light of Inflammatory Markers

**DOI:** 10.3390/diagnostics14141546

**Published:** 2024-07-17

**Authors:** Orçun Barkay, Faruk Karakeçili, Umut Devrim Binay, Sümeyye Akyüz

**Affiliations:** 1 Department of Infectious Diseases and Clinical Microbiology, Faculty of Medicine, Erzincan Binali Yıldırım University, 24100 Erzincan, Türkiye; drfarukkarakecili@hotmail.com (F.K.); devrimbinay@hotmail.com (U.D.B.); 2 Department of Medical Microbiology, Faculty of Medicine, Erzincan Binali Yıldırım University, 24100 Erzincan, Türkiye; sumeyye_sfl@hotmail.com

**Keywords:** brucellosis, Coombs gel test, ELISA, NLR, PLR, Rose Bengal, sacroiliitis SII, SIRI, spondylodiscitis

## Abstract

Background: Brucellosis is a zoonotic infectious disease. It is estimated that the number of cases reported today is much less than the actual number. We still have difficulty in diagnosing the disease and its organ involvement. In this sense, new approaches that can be useful in clinical practice are required, and we aimed to evaluate this situation in our study. Methods: 171 of 213 patients followed in our center between January 2021 and April 2024 were included in the study. A total of 150 patients were included in the study as a control group. Rose Bengal test (RBT), Coombs gel test (CGT), enzyme-linked immunosorbent assay (ELISA), and automated blood culture were used for diagnosing brucellosis. Complete blood count, sedimentation, C-reactive protein, and biochemical parameters were obtained. Inflammation markers such as neutrophil–lymphocyte ratio, platelet–lymphocyte ratio, systemic immune-inflammation index, and systemic inflammation response index were calculated. Results: The most successful results in the diagnosis were ELISA (89.4%), RBT (88.3%), CGT (83%), and blood culture (34.8%). For diagnosing sacroiliitis and spondylodiscitis, instead of resorting to expensive methods like magnetic resonance, a combination of ELISA positivity with elevated acute phase reactants and inflammatory markers could be significantly instructive. Conclusions: Optimizing diagnostic algorithms and exploring novel diagnostic approaches, such as inflammatory markers, hold promise for improving diagnosis and management.

## 1. Introduction

Brucellosis is a zoonotic infectious disease that has a low mortality rate, but it may cause high morbidity that can affect all systems in humans. The causative agents are bacteria of the genus *Brucella*. This genus consists of an increasing number of species infecting a variety of mammals as primary hosts, such as bovine (*B. abortus*), caprine (*B. melitensis*), swine (*B. suis*), ovine (*B. ovis*), camels, elk, bison (*B. abortus*), canine (*B. canis*), rodents (*B. neotomae*, *B. microti*), monkeys (*B. papionis*), marine mammals such as seals, dolphins and whales (*B. pinnipidialis* and *B. ceti*). The only ones that affect humans are *B. abortus*, *melitensis*, *suis*, and *canis*. It can be transmitted to humans directly or indirectly from infected animals or their products. The main route of transmission is the consumption of products made from raw milk [1,2,3]. The disease can be seen in all regions of the world and is also known as Malta Fever, Mediterranean Fever, Bang’s Disease, Gibraltar Fever, Undulant Fever, Goat Fever, and Rock Fever. Approximately 500,000 cases of human brucellosis are reported annually. It is believed that the actual number of cases is much higher than the reported figures [4]. In the European Union region, only 199 cases were detected in 2022 (the notification rate in the EU/EEA was 0.04 cases per 100,000 population.). Mediterranean countries, including Turkey, as well as North and East Africa, South and Central Asia, Middle Eastern countries, and Central and South America, are considered hyperendemic regions [5]. The most recent data for brucellosis in our country, Turkey, is from the year 2019, and according to the data of the Turkish Public Health Institution, in 2019, the number of brucellosis cases in Turkey was 10,244, with a morbidity rate of 12.32 per 100,000 with the highest incidence in the Southeastern and Eastern Anatolia regions where animal husbandry is more common [6,7]. The province of Erzincan, where our university hospital is located, is in the Eastern Anatolia Region. In the past three years, 213 new cases have been detected in our province alone. Considering that disease reporting is still not at a sufficient level in our country, the actual prevalence of brucellosis is likely much higher than estimated. Therefore, brucellosis remains a public health issue in our country [8,9].

Since it is an intracellular microorganism, it can affect various organs and systems such as the central nervous system (meningitis, encephalitis, etc.), cardiovascular system (endocarditis, etc.), hematopoietic system (pancytopenia), musculoskeletal system (myositis, spondylodiscitis, sacroileitis, septic arthritis, etc.), urogenital system (nephritis, orchitis, etc.), and due to this situation various laboratory findings can be found. Diagnosis of brucellosis and its system involvements, which can cause morbidity and mortality, is still challenging [10,11].

In our study, we aimed to compare the Rose Bengal test (RBT), Coombs gel test (CGT), enzyme-linked immunosorbent assay (ELISA), and blood culture used in brucellosis patients in the light of acute phase reactants (white blood cell count (WBC), sedimentation, C-reactive protein (CRP)), inflammatory parameters (neutrophil–lymphocyte ratio (NLR), platelet–lymphocyte ratio (PLR), systemic immune-inflammation index (SII), systemic inflammation response index (SIRI)), and biochemical parameters, and to determine the impact of this comparison on clinical practice.

## 2. Materials and Methods

### 2.1. Study Design

#### 2.1.1. Patient Selection

This retrospective case–control study was conducted at the Department of Infectious Diseases and Clinical Microbiology, Erzincan University Faculty of Medicine, Erzincan, Turkey. From January 2021 to April 2024, 172 out of 213 patients who presented to our hospital with clinical symptoms compatible with brucellosis and were diagnosed based on test results were included in the study. Forty-one patients were excluded due to comorbidities, additional bacterial infections, pregnancy, additional medication use, or other conditions that could affect inflammation-related data. The control group included 150 patients who visited our outpatient clinic for various reasons and had no conditions affecting inflammation.

#### 2.1.2. Patient Data

Demographic data, season of presentation, clinical type at the time of presentation (acute, subacute, chronic), and organ involvement at the time of presentation (sacroiliitis, spondylodiscitis, septic arthritis of the knee, hip, and finger joints, orchitis, meningitis, myositis, psoas abscess) were recorded. Patients with complaints lasting two months or less were considered acute, 2–12 months subacute, and more than one year chronic.

#### 2.1.3. Diagnostic Tests

In our study, the following tests were used for diagnosing brucellosis: Rose Bengal plate agglutination test (VIRCELL^®^, Granada, Spain), Coombs gel test (REDCELL^®^, Istanbul, Turkey), ELISA (Anti-Brucella abortus IgM and Anti-Brucella abortus Brucella IgG (Euroimmun AG^®^, Lübeck, Germany)), and blood culture (BacT/ALERT^®^ automated blood culture system (bioMérieux, Marcy l’Etoile, France)). Manufacturer of each test defined the sensitivity and specificity of RBT, CGT, and ELISA as 99–97.6%, 100–98.6%, 96.7–97.9%, respectively. Blood cultures were collected in 2 sets (2 aerobic and 2 anaerobic medium). A single culture positivity was considered sufficient. Samples from patients with focal disease were obtained by percutaneous aspiration and they were cultured on non-specific blood agar.

#### 2.1.4. Additional Tests

Complete blood count values (WBC, neutrophil, lymphocyte, monocyte, hemoglobin, platelet levels), specific inflammation markers such as NLR, PLR, SII, and SIRI, as well as biochemical parameters potentially related to organ involvement (blood urea nitrogen (BUN), creatinine, aspartate aminotransferase (AST), alanine aminotransferase (ALT)) were recorded.

#### 2.1.5. Inflammatory Markers

NLR was calculated as the neutrophil count/lymphocyte count; PLR as the platelet count/lymphocyte count; SII as (neutrophil count × platelet count)/lymphocyte count; and SIRI as (neutrophil count × monocyte count)/lymphocyte count.

### 2.2. Statistical Analyses

Descriptive statistics of the measurements were calculated as mean, standard deviation (SD), median, 25th and 75th quartiles, number, and % frequencies. The compliance of numerical type features obtained by measurement with normal distribution was examined with the Shapiro–Wilks test and it was determined that all variables did not show normal distribution. Differences between control and brucellosis patient groups in terms of numerical characteristics and various groups of patients were compared using the Mann–Whitney U test or Kruskal–Wallis test. The source of significant differences in more than two groups was determined by post hoc Dunn’s test. Relationships between categorical features were examined with Pearson chi-square or Fisher–Freeman exact test. The diagnostic success of the tests was compared with the *t*-test for the difference in proportions. Statistical significance level was accepted as *p* ≤ 0.05. SPSS (ver. 23) program was used in the calculations.

### 2.3. Ethical Approval

Ethical approval was gained from Ethics Committee of Erzincan Binali Yıldırım University (Number: 2024-05/04; Date: 4 April 2024). Informed consent was not obtained as it was a retrospective study.

## 3. Results

In this study, a total of 321 individuals were included, consisting of 171 patients diagnosed with brucellosis, of whom 95 (55.6%) were male, and 150 control subjects, of whom 74 (49.3%) were male. The mean age of brucellosis patients was 46.9 ± 15.03 years, and the mean age of the control group was 47.3 ± 15.03 years, with no significant difference observed between the two groups in terms of age (*p* = 0.734). The average age of male patients diagnosed with brucellosis was 45.5 ± 15.4, while the average age of females was 48.7 ± 14.2. Descriptive values for categorical characteristics of patients diagnosed with brucellosis were presented in Table 1.

The RBT was administered to all patients (*n* = 171) as a screening test, with 151 (88.3%) testing positive. Of the 20 patients who were found to be negative, 9 were in the acute phase, 6 were in the subacute phase, and 5 were in the chronic phase at the time of detection. The Coombs gel test was also performed on all patients (*n* = 171), with 142 (83%) testing positive when a titer of ≥1/160 was considered positive, while among the 19 (17%) patients who tested negative, 8 (4.7%) had a titer of 1/80. Among these patients, five tested positive on ELISA. It is noteworthy that 10 of these 19 patients whose results were found to be negative were in the acute phase. Further examination of the 142 patients who tested positive on the CGT revealed titers of 1/160 in 46 individuals (26.9%), 1/320 in 93 individuals (54.4%), and 1/640 in 3 individuals (1.8%). ELISA testing was performed on 85 patients, with 76 (89.4%) testing positive. Among these 76 patients, 19 tested negative on the CGT. Of the 9 patients who tested negative on ELISA, 8 tested positive on the CGT (8 patients at 1/320, 1 patient at 1/160).

Seventy-five patients diagnosed with brucellosis were followed up as inpatients; blood cultures, considered the gold standard for diagnosing brucellosis, were obtained from 66 of these patients, with growth detected in 23 (34.8%). Seven patients were followed up for septic arthritis in the knee joint, and one patient for septic arthritis in the hip joint, with all eight patients showing growth in joint fluid cultures. One patient was followed up for septic arthritis in the finger joint, although joint fluid culture was not obtained. Brucella was also isolated from an abscess culture obtained from a patient with intra-abdominal abscess.

According to these results, it can be said that the tests that give the most successful results in the diagnosis of brucellosis are the ELISA test (89.4%), RBT (88.3%), CGT (83%), and blood culture (34.8%), respectively. However, no statistically significant difference was found in the positivity rates among these tests.

No significant relationship was observed between the clinical type at the time of diagnosis and the results of the RBT, CGT, and ELISA. Accordingly, the distribution of acute, subacute, and chronic disease rates was found to be similar among cases with negative and positive results for these three tests.

Significantly higher rates of acute onset were observed among those with positive blood cultures, while significantly higher rates of chronic onset were observed among those with negative blood cultures (*p* = 0.05). It was determined that 82 (47.9%) cases were in the acute phase of the disease. RBT was studied in all patients and was found positive in 73 (89%). CGT was performed in all patients and was found positive in 72 (87.8%). ELISA was studied in 50 patients and was found positive in 45 (90%). Blood cultures were studied in 45 patients during this period and were found positive in 19 of them (42.2%). ELISA stands out as the most successful test in the acute period, but no statistically significant results were obtained.

When the comparative results of diagnostic tests for detecting the presence of sacroiliitis were examined, no significant relationship was found between any test result and the presence of sacroiliitis. Similar rates of sacroiliitis were found among cases with negative and positive results for all four tests.

Similarly, when the comparative results of diagnostic tests for detecting the presence of spondylodiscitis were examined, no significant relationship was found between the results of the RBT, CGT, and blood culture, and the presence of spondylodiscitis. Similar rates of spondylodiscitis were found among cases with negative and positive results for these three tests. Only in cases with positive ELISA tests, the presence of spondylodiscitis was significantly higher (*p* = 0.05).

When evaluating the comparative results of diagnostic tests for detecting the presence of septic arthritis in the knee, orchitis, and meningitis, no significant relationship was found between any test result and these conditions. Similar rates of these conditions were found among cases with negative and positive results for all four tests.

Only one case each of septic arthritis in the hip, arthritis in the finger joint, myositis, and psoas abscess were present, hence their relationship with test results could not be assessed.

Descriptive values for acute phase reactants, inflammatory markers, and biochemical parameters according to the clinical type at the time of diagnosis are presented in Table 2. Platelet levels, sedimentation rate, CRP levels, AST-ALT levels, PLR, and SII averages were significantly higher in acute cases compared to chronic cases. No other significant differences were found.

No significant relationship was found between gender distribution and the clinical type at the time of diagnosis, as well as the presence of organ involvement. Likewise, there was no significant change in the clinical type at the time of diagnosis and the presence of organ involvement according to the season of application.

When examining the descriptive values of acute phase reactants, inflammatory markers, and biochemical parameters in patients with and without sacroiliitis, it was found that CRP, AST-ALT, and NLR levels were high in patients with sacroiliitis, but only the average AST level was significantly higher (*p* = 0.009). No other significant differences were observed.

Similarly, when examining the descriptive values of acute phase reactants, inflammatory markers, and biochemical parameters in patients with and without spondylodiscitis, it was found that WBC, sedimentation rate, CRP, NLR, PLR, SII, and SIRI levels were high in patients with spondylodiscitis, but only the average sedimentation rate and CRP level were significantly higher (*p* = 0.043, *p* = 0.006). No other significant differences were observed.

When examining the descriptive values of acute phase reactants, inflammatory markers, and biochemical parameters in patients with and without septic arthritis in the knee, only the average hemoglobin level was significantly lower in patients with septic arthritis in the knee (*p* = 0.03). No other significant differences were observed.

In patients with and without orchitis, when examining the descriptive values of acute phase reactants, inflammatory markers, and biochemical parameters, it was found that WBC, neutrophil, lymphocyte, monocyte, hemoglobin, platelet, CRP, NLR, SII, SIRI, creatinine, AST-ALT levels were high in patients with orchitis, but only the average hemoglobin and AST-ALT levels were significantly higher (*p* = 0.008, *p* = 0.034, *p* = 0.04). No other significant differences were observed.

When patients were grouped as those with and without organ involvement and the descriptive values of acute phase reactants, inflammatory markers, and biochemical parameters were examined, it was found that in patients with organ involvement, all acute phase reactants (WBC, neutrophil, lymphocyte, monocyte, sedimentation rate, CRP), platelet levels, and all inflammatory markers (NLR, PLR, SII, SIRI) were high. However, only the average CRP and AST levels were significantly higher (*p* = 0.006, *p* = 0.012). No other significant differences were observed (Table 3).

The descriptive statistics of numerical features and the comparison results between patients diagnosed with brucellosis and the control group are provided in Table 4. Neutrophil count, lymphocyte count, monocyte count, hemoglobin level, sedimentation rate, CRP level, SII, and SIRI levels were found to be higher in the brucellosis group compared to the control group, but no significant difference was found except for hemoglobin level and sedimentation rate parameters.

## 4. Discussion

Brucellosis is a disease that can affect individuals of all ages. The gender distribution is generally equal. The age and gender distribution of the patients in our study are similar to those reported in other studies [4,12,13]. In a study conducted in our region, it was observed that the majority of individuals engaged in livestock farming were in the 41–50 age range as in our study [14]. Therefore, it is an expected finding that patients in studies conducted to date, including our study, are typically within this age group.

A definitive diagnosis of brucellosis is the culture of blood, bone marrow, tissues, or retained body fluids such as joint fluid. Automated blood culture systems provide faster results than conventional culture methods. Because of the problems like low sensitivity, requiring a long incubation period, potential risk of laboratory infection, difficulties at the isolation of the agent (different disease phases (acute, subacute, chronic), antibiotic pre-use); serological tests and compatible clinical findings are commonly used in practice instead of blood culture which is considered as the gold standard for diagnosis [15,16,17,18,19]. Antibody tests are also widely used because of these reasons [16,18,19,20,21,22]. Blocking antibodies can be found in chronic cases, which can specifically bind to the antigen without visible agglutination. The presence of blocking antibodies in the serum can be shown by CGT or BrucellaCapt test [18,19,21,22,23]. Alternative methods, such as ELISA can also be used [24].

We know that serological tests sometimes may have false results, in particular in the case of cross-reactions with other Gram-negative bacteria such as *Escherichia coli*, *Yersinia enterocolitica*, and *Salmonella urbana* [25]. Therefore, we did not include patients who we suspected were infected or might have been infected with these bacteria in the study. We use automated blood culture systems at inpatient follow-up; and CGT and ELISA at in- and outpatient follow-up to have more accurate and faster results for diagnosis.

Among the diagnostic tests, RBT, used as a screening/rapid diagnostic test, has a high sensitivity (approximately 90%). Due to its nature as an antibody screening test, false negatives can be observed during the acute phases of the disease [7]. Indeed, in our study, the positivity rate of the RBT was found to be 88.3%. It was observed that the majority of patients with negative RBT results were in the acute and subacute patient groups. Similarly, false negatives can also be observed in the CGT during the acute phase. As a similar finding in our study, it was determined that the majority of patients with negative CGT results were in the acute phase. Although high rates of blood culture positivity are observed during this period, it is not a practical method for diagnosis. As noted in our study and the other studies, requesting the ELISA test for diagnosis during the acute phase seems to be more appropriate [24,26].

The positivity rates of the CGT have been found to vary across different studies, including ours [26]. This variation may be related to the clinical type of the disease. In our study, the positivity rate of blood cultures is higher compared to other studies [27,28]. Considering that culture positivity is more common in the acute phase, the higher number of patients in the acute phase in our study may explain this observation.

The sensitivity rates of the tests we found in our study were far below the rates given by the manufacturers. Since RBT and CGT are more observation-based tests, there may be laboratory-related false negativity, but we still believe that a re-evaluation should be made in this sense in line with these results.

In the literature, no other study has been found that shows a relationship between ELISA test positivity and the presence of spondylodiscitis. This finding places the ELISA test a step ahead of other tests. However, further studies in this area are still needed.

Due to the intracellular nature of the microorganism, it can affect various organs and systems, which causes a wide variety of clinical presentations. Because of this situation, the presenting symptoms of the infection are not pathognomonic; therefore, the disease can be easily confused with other medical conditions. As there are various clinical symptoms/findings, laboratory parameters may also vary in a broad range. Leukocytosis, leukocytopenia, anemia, thrombocytopenia, and thrombocytosis may be seen in whole blood count whereas it can be totally normal. Due to regional lymph node involvement, leukocytosis and elevated CRP can be detected, especially in the early stages of the infection. Elevated sedimentation rate may also be observed. The acute phase, then may progress to a subacute or chronic disease. In the later stages of the infection, pancytopenia may occur due to reticuloendothelial system involvement, and abnormalities in biochemical parameters (such as elevated liver function tests) may arise due to the involvement of various organs [16,17,29,30,31,32,33].

Although not statistically significant in the acute phase of brucellosis, the average neutrophil count was also found to be elevated in our study. In this context, the high platelet count in this group was considered more likely to be reactive thrombocytosis. Our findings are consistent with the general information given above.

In contemporary medical practice, inflammation parameters such as NLR, PLR, SII, and SIRI are frequently employed in the diagnosis and course monitoring of various diseases [34,35,36,37]. However, there is a lack of studies in the literature regarding the utilization of these parameters in brucellosis.

Although a significant purulent inflammation is generally not expected in brucellosis due to the intracellular structure of the pathogen, we have performed something that has not yet been found in the literature and compared these markers between the patient group diagnosed with brucellosis and the control group. In this regard, our study also found no marked difference in inflammation between these groups compatible with the general knowledge. However, it is entirely natural for acute phase reactants and inflammatory markers to be elevated in cases of organ involvement due to brucellosis as the literature says [38,39]. Due to our findings, we suggest that, for diagnosing sacroiliitis and spondylodiscitis, instead of resorting to expensive methods like magnetic resonance imaging for every patient, using a combination of ELISA positivity with elevated acute phase reactants and inflammatory markers could be significantly instructive. While increases in AST and ALT generally indicate reticuloendothelial system involvement, our study’s findings may also suggest organ involvement.

## 5. Conclusions

Diagnosing brucellosis is complex, needing both clinical and laboratory methods. Our study highlights the sensitivity of various diagnostic tests and the need for multiple methods for accurate detection. Brucellosis remains a significant issue in endemic areas, calling for better surveillance and control. Despite available tests, the disease’s prevalence may be underestimated, necessitating continued screening. Future efforts should focus on optimizing diagnostic methods and exploring new approaches, like inflammatory markers, to improve diagnosis and management, ultimately reducing the disease’s impact and enhancing patient outcomes globally.

## Figures and Tables

**Table 1:** Descriptive values for categorical characteristics of patients diagnosed with brucellosis.

		Brucellosis	
		*n*	%
Application season	Spring	40	23.4
	Summer	45	26.3
	Autumn	42	24.6
	Winter	44	25.7
Rose Bengal test	Negative	20	11.7
	Positive	151	88.3
Coombs gel test	Negative	21	12.3
	1/80	8	4.7
	1/160	46	26.9
	1/320	93	54.4
	1/640	3	1.7
Coombs gel test	Negative	29	17.0
	Positive	142	83.0
ELISA Test	Negative	9	10.6
	Positive	76	89.4
Blood culture growth	Negative	43	65.2
	Positive	23	34.8
Additional culture growth	Knee joint fluid	7	77.8
	Hip joint fluid	1	11.1
	Intraabdominal abscess	1	11.1
Follow-up	Outpatient	96	56.1
	Inpatient	75	43.9
Clinical type	Acute	82	48.0
	Subacute	39	22.8
	Chronic	50	29.2
Sacroiliitis	No	125	73.1
	Yes	46	26.9
Spondylodiscitis	No	140	81.9
	Yes	31	18.1
Septic arthritis of the knee	No	164	95.9
	Yes	7	4.1
Septic arthritis of the hip	No	170	99.4
	Yes	1	0.6
Septic arthritis of the finger	No	170	99.4
	Yes	1	0.6
Orchitis	No	165	96.5
	Yes	6	3.5
Meningitis	No	168	98.2
	Yes	3	1.8
Myositis	No	170	99.4
	Yes	1	0.6
Psoas abscess	No	170	99.4
	Yes	1	0.6
Organ involvement	No	96	56.1
	Yes	75	43.9

**Table 2:** Descriptive values of acute phase reactants, biochemical parameters, and inflammation markers according to the clinical type of the disease at the time of detection.

	Acute			Subacute			Chronic			*p*
	*n*	Mean	SD	*n*	Mean	SD	*n*	Mean	SD	
Age	82	45.39	16.09	39	50.03	12.55	50	47.20	14.89	0.238
WBC (/mm^3^)	82	7500.00	2738.40	39	7247.18	2358.02	50	6659.60	1800.24	0.275
Neutrophil (/mm^3^)	82	4658.78	2441.47	39	4316.15	1916.39	50	3928.20	1618.81	0.277
Lymphocyte (/mm^3^)	82	2073.41	712.16	39	2217.18	911.88	50	2189.80	737.03	0.779
Monocyte (/mm^3^)	82	604.15	260.42	39	552.56	197.36	50	561.40	198.60	0.711
Hemoglobin (g/dL)	82	13.80	1.60	39	13.74	1.86	50	14.09	2.21	0.647
Platelet (/mm^3^)	82	270,609.7 ^a^	93,427.9	39	245,205 ^ab^	88,528.1	50	223,320.0 ^b^	60,294.6	0.007
Sedimentation (mm/h)	82	20.39 ^a^	18.72	39	15.31 ^ab^	16.53	50	11.58 ^b^	9.30	0.017
CRP (mg/L)	82	27.37 ^a^	32.22	39	10.90 ^b^	13.37	50	7.37 ^b^	10.22	0.001
BUN (mg/dL)	82	31.70	10.30	39	30.83	8.80	50	29.09	9.98	0.358
Creatinine (mg/dL)	82	0.85	0.16	39	0.85	0.16	50	0.83	0.16	0.712
AST (IU/L)	82	34.54 ^a^	32.49	39	22.56 ^b^	6.80	50	25.96 ^b^	15.09	0.020
ALT (IU/L)	82	41.24 ^a^	42.17	39	22.49 ^b^	11.97	50	25.58 ^b^	21.74	0.009
NLR	82	2.55	1.75	39	2.25	1.35	50	1.97	1.02	0.250
PLR	82	144.80 ^a^	70.79	39	124.67 ^b^	63.11	50	113.75 ^b^	49.16	0.010
SII	82	705,168.0 ^a^	624,627.1	39	574,522.9 ^ab^	567,908.0	50	450,318.7 ^b^	308,421.6	0.004
SIRI	82	1738.4	1839.8	39	1220.4	782.9	50	1116.7	715.6	0.375

WBC: white blood count, CRP: C-reactive protein, BUN: blood urea nitrogen, AST: aspartate aminotransferase, ALT: alanine aminotransferase, NLR: neutrophil–lymphocyte ratio, PLR: platelet–lymphocyte ratio, SII: systemic immune-inflammation index, SIRI: systemic inflammation response index, ^a,ab,b^: Explains whether the compared parameters show significant differences or not (while parameters with different letters show a significant difference, those with the same letter or a common letter do not show a significant difference).

**Table 3:** Descriptive values of acute phase reactants, biochemical parameters, and inflammation markers in patients with and without organ involvement.

	Organ Involvement	*n*	Mean	SD	*p*
WBC (/mm^3^)	Yes	75	7309.9	2618.9	0.878
	No	96	7108.1	2271.9	
Neutrophil (/mm^3^)	Yes	75	4477.6	2219.5	0.549
	No	96	4280.6	2055.3	
Lymphocyte (/mm^3^)	Yes	75	2142.7	841.3	0.710
	No	96	2138.3	708.1	
Monocyte (/mm^3^)	Yes	75	598.3	245.1	0.691
	No	96	565.5	217.9	
Hemoglobin (g/dL)	Yes	75	13.8	1.9	0.882
	No	96	13.9	1.8	
Platelet (/mm^3^)	Yes	75	261,280.0	94,185.6	0.125
	No	96	242,947.9	78,373.9	
Sedimentation (mm/h)	Yes	75	17.6	15.6	0.362
	No	96	15.9	17.0	
CRP (mg/L)	Yes	75	22.9	28.6	0.006
	No	96	13.7	22.2	
BUN (mg/dL)	Yes	75	32.0	9.2	0.143
	No	96	29.8	10.3	
Creatinine (mg/dL)	Yes	75	0.9	0.1	0.725
	No	96	0.8	0.2	
AST (IU/L)	Yes	75	30.8	19.1	0.012
	No	96	28.1	28.3	
ALT (IU/L)	Yes	75	36.1	38.2	0.143
	No	96	29.4	28.2	
NLR	Yes	75	2.4	1.6	0.462
	No	96	2.2	1.4	
PLR	Yes	75	137.1	64.3	0.146
	No	96	126.5	64.7	
SII	Yes	75	652,551.7	611,644.5	0.184
	No	96	560,466.0	486,998.8	
SIRI	Yes	75	1486.6	1294.9	0.280
	No	96	1401.0	1495.8	

WBC: white blood count, CRP: C-reactive protein, BUN: blood urea nitrogen, AST: aspartate aminotransferase, ALT: alanine aminotransferase, NLR: neutrophil–lymphocyte ratio, PLR: platelet–lymphocyte ratio, SII: systemic immune-inflammation index, SIRI: systemic inflammation response index.

**Table 4:** Descriptive statistics of numerical features in the control and brucellosis groups.

	Group	*n*	Mean	SD	Percentiles			*p*
					25th	Median	75th	
WBC (/mm^3^)	Control	150	7282.7	1691.4	6097.5	7165.0	8502.5	0.113
	Brucellosis	171	7196.6	2424.8	5600.0	6800.0	8400.0	
Neutrophil (/mm^3^)	Control	150	4243.3	1382.8	3190.0	4170.0	5160.0	0.368
	Brucellosis	171	4367.0	2124.8	3090.0	3900.0	5120.0	
Lymphocyte (/mm^3^)	Control	150	2027.5	678.8	1530.0	1970.0	2490.0	0.249
	Brucellosis	171	2140.2	767.0	1550.0	2060.0	2640.0	
Monocyte (/mm^3^)	Control	150	552.1	218.7	400.0	520.0	692.5	0.545
	Brucellosis	171	579.9	230.1	430.0	520.0	700.0	
Hemoglobin (g/dL)	Control	150	13.3	1.6	11.9	13.0	14.3	0.001
	Brucellosis	171	13.9	1.8	12.8	13.9	15.1	
Platelet (/mm^3^)	Control	150	257,766	88,824	197,250	237,500	305,000	0.766
	Brucellosis	171	250,988	85,891	193,000	242,000	296,000	
Sedimentation (mm/h)	Control	150	11.1	7.5	5.0	10.0	16.0	0.020
	Brucellosis	171	16.7	16.4	5.0	12.0	22.0	0.113
CRP (mg/L)	Control	150	8.4	7.0	3.0	5.0	11.9	
	Brucellosis	171	17.8	25.5	3.0	4.1	24.5	0.368
NLR	Control	150	2.31	1.11	1.48	2.18	2.71	0.127
	Brucellosis	171	2.31	1.49	1.34	1.78	2.76	
PLR	Control	150	140.51	70.62	92.29	126.16	169.46	0.237
	Brucellosis	171	131.13	64.52	94.79	119.33	155.45	
SII	Control	150	595,330	403,695	353,000	531,395	730,501	0.148
	Brucellosis	171	600,854	545,420	322,859	440,770	696,974	
SIRI	Control	150	1235.94	697.32	746.99	1055.14	1598.43	0.533
	Brucellosis	171	1438.51	1407.86	616.66	1027.43	1585.39	

WBC: white blood count, CRP: C-reactive protein, NLR: neutrophil–lymphocyte ratio, PLR: platelet–lymphocyte ratio, SII: systemic immune-inflammation index, SIRI: systemic inflammation response index.

## Data Availability

Data are contained within the Supplementary Materials.

## Supplementary Materials

Supporting file including raw data can be downloaded at: https://zenodo.org/records/12753039 (accessed on 8 June 2024).
